# Soil-Transmitted Helminths in Children in a Remote Aboriginal Community in the Northern Territory: Hookworm is Rare but *Strongyloides stercoralis* and *Trichuris trichiura* Persist

**DOI:** 10.3390/tropicalmed2040051

**Published:** 2017-10-04

**Authors:** Deborah C. Holt, Jennifer Shield, Tegan M. Harris, Kate E. Mounsey, Kieran Aland, James S. McCarthy, Bart J. Currie, Therese M. Kearns

**Affiliations:** 1 Menzies School of Health Research, Charles Darwin University, Darwin NT 0811, Australia; deborah.holt@menzies.edu.au (D.C.H.); j.shield@latrobe.edu.au (J.S.); tegan.harris@menzies.edu.au (T.M.H.); bart.currie@menzies.edu.au (B.J.C.); 2 Department of Pharmacy and Applied Science, La Trobe University, Bendigo VIC 3550, Australia; 3 School of Health and Sports Science, University of the Sunshine Coast, Maroochydore QLD 4558, Australia; kmounsey@usc.edu.au; 4 Queensland Museum, South Brisbane QLD 4101, Australia; bioscreenoz@gmail.com; 5 QIMR Berghofer Medical Research Institute, Herston QLD 4006, Australia; j.mcarthy@uq.edu.au; 6 School of Medicine, University of Queensland, Brisbane QLD 4072, Australia

**Keywords:** *Strongyloides stercoralis*, strongyloidiasis, *Trichuris trichiura*, *Rodentolepis nana*, Northern Territory, Aboriginal

## Abstract

(1) Background: soil-transmitted helminths are a problem worldwide, largely affecting disadvantaged populations. The little data available indicates high rates of infection in some remote Aboriginal communities in Australia. Studies of helminths were carried out in the same remote community in the Northern Territory in 1994–1996 and 2010–2011; (2) Methods: fecal samples were collected from children aged <10 years and examined for helminths by direct smear microscopy. In the 2010–2011 study, some fecal samples were also analyzed by agar plate culture and PCR for *Strongyloides stercoralis* DNA. Serological analysis of fingerprick dried blood spots using a *S. stercoralis* NIE antigen was also conducted; (3) Results and Conclusions: a reduction in fecal samples positive for *S. stercoralis*, hookworm and *Trichuris trichiura* was seen between the studies in 1994–1996 and 2010–2011, likely reflecting public health measures undertaken in the region to reduce intestinal helminths. Comparison of methods to detect *S. stercoralis* showed that PCR of fecal samples and serological testing of dried blood spots was at least as sensitive as direct smear microscopy and agar plate culture. These methods have advantages for use in remote field studies.

## 1. Introduction

Soil-transmitted helminths are a worldwide problem generally affecting poor and vulnerable populations [1]. In Australia, there is a paucity of studies documenting the prevalence of soil-transmitted helminths due to the difficulties in diagnosing infection in communities, and timely transport to the nearest diagnostic laboratory. A cross-sectional survey in a remote Aboriginal community in the north of Western Australia in 1992 found hookworm infection in 77% of participants with the highest prevalence in children aged 5–14 years (93%) [2]. In a remote community in the Northern Territory (NT), a study in the mid-1990s of children and adults documented high rates of infection with hookworm, *Strongyloides stercoralis*, *Trichuris trichiura* and *Rodentolepis* (*Hymenolepis*) *nana* [3]. More recent surveys of *S. stercoralis* prevalence indicate that it is endemic in many northern Australian Aboriginal communities [4,5,6].

Currently, there is no gold standard test for diagnosing *S. stercoralis* [7,8]. Stool examination underestimates the prevalence of the parasite in population-based studies, while serological testing gives a higher prevalence [9]. When examining stools, the larval density is often low and output sporadic, resulting in variation in detection between samples in the same individuals [10]. Routine direct smear microscopy of single stool specimens has a low sensitivity in chronic cases and can fail to detect larvae in up to 70% of chronic infections [7,10]. The use of the agar culture plate technique has improved detection in chronic *S. stercoralis*, with a sensitivity of 96% when compared with direct fecal smear, formalin-ethyl acetate concentration and Harada-Mori filter paper culture [7]. A practical problem for the agar plate technique is that viable larvae are required for culture, which can be problematic for specimens that involve long delays (transport or otherwise) in reaching the laboratory. Serological examination for *S. stercoralis* antibodies improves detection in those with chronic infection. However, it may not readily detect those with acute infection as the prepatent period can be up to 28 days [8,11].

A study in a remote NT Aboriginal community in 1994–1996 revealed a high level of intestinal helminths by formol-ether concentration of fecal samples [3]. Concern of residents in this community about high rates of *S. stercoralis* infection resulted in a project in 2010–2011 to investigate the utility of mass drug administration to reduce the endemic prevalence of *S. stercoralis* and scabies [6,12]. The aim of this study was to compare the prevalence of intestinal parasites identified by direct smear microscopy between the 1994–1996 and 2010–2011 studies. In addition, due to the logistical challenges of handling and processing fecal samples during remote field studies, we also investigated the utility of alternative methods of *S. stercoralis* diagnosis during the 2010–2011 study. Serological testing of eluted dried blood spots and PCR were shown to be at least as sensitive as microscopy and culture, and have considerable advantages for use in remote community settings.

## 2. Materials and Methods 

### 2.1. Sample Population

Samples were collected from consenting participants during two separate projects in a remote Aboriginal community with an estimated population of 2000, located 550 km east of Darwin, NT Australia. Ninety-four percent of the resident population are Australian Aboriginal with an average of 6.3 members per household, and 19.9% of the population are aged <10 years [13]. Each project received ethical approval from the Human Research Ethics Committee of the Northern Territory Department of Health and Menzies School of Health Research (EC00153; approvals 94/19 and 09/34).

### 2.2. Fecal Sample Collection and Processing

In the first study, surveys of intestinal parasites were conducted in the community from 1994–1996 [3]. Fecal samples were collected into disposable plastic containers by a parent or carer and collected by the researchers the following morning. Direct smears for the identification of intestinal parasites were undertaken in a field laboratory. Quantitative formol-ether counts conducted on fecal samples preserved in 4% formaldehyde were previously reported [3]. Treatment was administered by the local primary health care service and was not recorded as part of the study.

The 2010–2011 study consisted of two population censuses and mass drug administrations (MDAs) conducted 12 months apart in a staged roll-out. The full study design has been previously reported [6,12]. Fecal samples were collected from children by a parent or carer into disposable plastic containers and returned to the researchers. Direct smear microscopy on approximately 0.005 g of feces was performed on the majority of samples on site in a field laboratory within four hours of receipt. A small number of samples from 2011 were fixed in SAF (sodium acetate, acetic acid, formalin) and transported by aircraft to a commercial pathology laboratory for microscopic analysis the day after receipt. Only the results of the first fecal sample collected from each participant aged <10 years are reported here.

Fisher exact probability tests were performed at the VassarStats website [14].

### 2.3. Agar Plate Culture

For samples with sufficient fecal matter, agar plate culture was conducted, based on the method of Garcia [15], except that nutrient-deficient Mueller-Hinton agar plates were used, with the aim of reducing fungal growth. Specifically, a patch of ~2 cm diameter of feces was applied to the center of a nutrient-deficient Mueller-Hinton agar plate, sealed, and incubated at room temperature (~25 °C) overnight. The plates were then transported by aircraft to the research laboratory the following day in a foam container with a sweated ice brick to maintain a temperature of 17–25 °C. The plates were maintained in the research laboratory at 25 °C and examined macroscopically for larval tracks marked by bacterial colonies daily for up to five days after plating. Once tracks were observed, or after five days had elapsed, the plate was flooded with 10% formalin for five minutes, the liquid aspirated and centrifuged at 500× *g*, and the sediment examined microscopically for *S. stercoralis* and hookworm infective larvae.

### 2.4. Fecal DNA Extraction and PCR

Any remaining fecal material was transported to the research laboratory, where it was stored in ethanol at −20 °C. DNA extraction using up to 30 mg of stored fecal samples was performed using a PowerSoil^®^ DNA isolation kit (MoBio Laboratories Inc, Carlsbad, CA, USA), according to the manufacturer’s instructions. The PowerSoil kit is designed for use with complex samples and has been shown to be superior to four other DNA extraction methods in terms of sensitivity and ease of use, for extraction and detection of *Strongyloides ratti* DNA in spiked human stool samples [16]. DNA samples were tested using a published real-time PCR based method for the detection of *S. stercoralis* 18S rDNA [17]. Due to a high percentage of positive samples in 2010 that were detected using this method (34/39), the real-time PCR products were analyzed by agarose gel electrophoresis. Many samples did not have the correct 101 bp product, but had a smaller DNA fragment, possibly primer dimer, that presumably reacted with the probe to produce fluorescence in the real-time PCR. As a result, we designed an alternative forward primer (Stro18S-altF 5’ GGGCCGGACACTATAAGGAT 3’), which produced a 471 bp product with the published Stro18S-1630R primer (5’ TGCCTCTGGATATTGCTCAGTTC 3’) [17]. The original Stro18S-1530F and Stro18S-1630R primer set was shown to be highly specific for *S. stercoralis* [17]; however, the specificity when using the alternative forward primer designed here was not systematically tested. End-point PCR using this alternative primer combination was conducted using 2 µL of DNA extraction, 20 pmol each primer, 100 µM dNTPs, 1.5 mM Mg^2+^ and 1 U *Taq* polymerase in a total volume of 20 µL. Cycling conditions were 35 cycles of 95 °C for 30 s, 58 °C for 30 s, and 72 °C 30 s. A positive control plasmid was constructed by cloning a PCR product obtained from an agar plate culture-positive sample into pBlueScript II SK. The efficiency of the PCR was optimized using the plasmid control and a culture-positive fecal sample.

### 2.5. Blood Spot Collection and Serological Testing

Dried blood spots were collected by fingerprick onto filter paper cards, air dried and stored in zip-lock bags with silica desiccant at 4–8 °C. Serum was eluted from the dried blood spots and analyzed by ELISA using a recombinant *S. stercoralis* NIE antigen [18] as previously reported [19]. Briefly, dried blood spots were eluted in 150 µL phosphate buffered saline and 0.05% Tween 20 (PBS-T) overnight at room temperature. A 1:500 dilution of this eluate was used in the NIE ELISA. Plates were coated with 100 µL of 125 ng/mL NIE antigen, and blocked with 5% skim milk powder in PBS-T. Dried blood spot elutions were added and incubated at 37 °C for two hours. Alkaline phosphatase conjugated goat anti-human IgG was used as the secondary antibody at a dilution of 1:2500. Plates were developed with phosphatase substrate and optical density read at 405 nm. The assay was initially established and validated using a panel of sera from participants that were either positive or negative for *S. stercoralis* by fecal culture. Each ELISA assay included positive and negative control dried blood spots, which were used to validate the assay and normalize optical density (OD) results [19]. Normalized ODs were calculated by the ratio of test sample OD to positive control dried blood spot OD. The result of the first sample collected from each participant is reported here.

## 3. Results

### 3.1. Comparison of Intestinal Parasites Identified in Children <10 Years in 1994–1996 and 2010–2011

Fecal samples were collected from children aged <10 years and examined by direct smear. Samples were collected from 84 participants in 1994–1996 (mean age 5.6 years) and 85 children in 2010–2011 (mean age 3.7 years). The percentage of samples positive for hookworm and *T. trichiura* in 2010–2011 was significantly less than that reported in 1994–1996 (*p* = 0.002 and 0.012 respectively). The percentage of samples positive for *S. stercoralis* also reduced, however *R. nana* remained unchanged (Table 1). The mean number of intestinal parasites identified per fecal sample was 1.5 in 1994–1996 and 2 in 2010–2011 (data not shown).

### 3.2. Comparison of Diagnostic Methods for S. stercoralis

**Figure 1:**
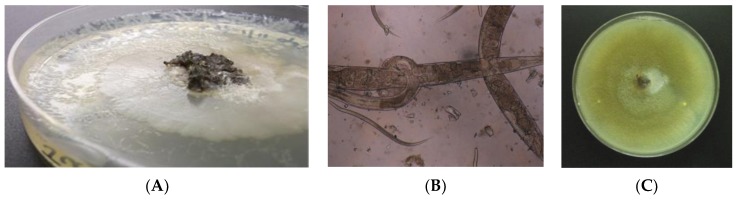
Identification of *S. stercoralis* larvae by agar plate culture and formalin sedimentation: (**A**) Bacterial growth on Mueller-Hinton agar in tracks made by helminth larvae; (**B**) *S. stercoralis* adults and filariform larvae in formalin sediment of agar culture; (**C**) Fungal overgrowth may have obscured larval tracks on some plates.

In addition to the direct smears, in the 2010–2011 study agar plate culture for helminth larvae, and PCR for *S. stercoralis* DNA was conducted on a subset of fecal samples, and serology was performed on sera eluted from dried blood spots (Table 2). *S. stercoralis* larvae were detected in five of 77 (6.5%) samples examined by agar plate culture and formalin sedimentation (Figure 1). Four of these samples were positive for *S. stercoralis* by direct smear, while one was negative. No hookworm larvae were detected. End point PCR for *S. stercoralis* 18S rDNA was positive for six (7.2%) of 83 samples tested, which included the five agar plate-positive samples. The percentage of samples considered positive by serology was higher than for the other methods with 25 (16.2%) of the dried blood spot samples considered positive.

There were 28 blood spot samples for which a fecal sample had also been analyzed by agar plate culture at the same time point. Of these, 20 participants were negative for both methods, and five were positive by serology but negative by culture. It is unknown if these children had recent infections or if results were false positives, as children are rarely tested for *Strongyloides* in this setting. For three participants who were positive by agar plate culture and had blood spots collected, one was also positive by serology, but the other two were negative by serology. This indicates that this serological method may not detect some acute infections.

## 4. Discussion

This is one of the first NT studies to examine and compare helminth infections in a remote Aboriginal community using three different diagnostic methods. Aboriginal Australians are traditionally hunter-gatherer societies, and there has been a rapid and often problematic transition to permanent settlement since European colonization. Aboriginal Australians have a higher burden of disease compared with non-Aboriginal Australians, with the largest difference seen in remote communities [20]. In remote community settings in northern Australia, government programs concentrating on the provision of infrastructure alone have been shown to have limited impact on community-level crowding and hygiene [21] or common childhood infectious diseases [22,23].

The introduction of a deworming program using albendazole in 1995 [24] is likely to have contributed to the reduction in samples positive for *S. stercoralis* (not statistically significant), hookworm and *T. trichiura* between the two study periods. The observed low rate of hookworm in 2010–2011 is supported by a downward trend in hookworm infections seen in a hospital-based study conducted during the same time period, which reported 14 cases per 100,000 in 2002 and 2.2 cases per 100,000 in 2012 [25]. The percentage of fecal samples positive for *S. stercoralis* dropped from 13.1% in 1994–1996 to 4.7% in 2010–2011, which reflects an overall reduction in the NT [26]. The single-dose albendazole used in the NT deworming program may have had a partial effect despite being lower than the recommended dose for treating *S. stercoralis* infection [26]. Serology on blood spots was positive for 16.2% of children tested in 2010–2011, higher than for fecal detection methods. The community-wide mass drug administration at the time of this study was shown to reduce the rate of seropositivity in treated participants 12 months later [6]. The reduction in participants positive for *T. trichiura* between the 1994–1996 study and the 2010–2011 study is consistent with overall data for the NT [27]; however, the percentage of participants positive for *T. trichiura* remained high in 2010–2011 at 48.2%. This may be due to the fact that single-dose albendazole reduces egg counts but has a low cure rate for *T. trichiura* infection [28,29,30]. The prevalence of *R. nana* infection remained unchanged between the study periods, consistent with a recent analysis of infections in the NT, which showed that infections were predominantly in Aboriginal children aged under 5 years [31]. Single-dose albendazole does not appear to produce a significant cure rate for *R. nana* [28] and the recommended treatment, praziquantel [32], is rarely stocked in health services in rural and remote communities.

As *S. stercoralis* larval output is often low and intermittent in chronic cases [10], a limitation of this study is the examination of a single fecal specimen for diagnosis. Agar plate culture and formalin sedimentation yielded only a single additional *S. stercoralis* positive sample compared with the direct smear method in 2010–2011, and this method is reported to have greater sensitivity than both direct smear and formol-ether concentration [33]. Fungal growth on the agar culture plates was common, and may have hindered the identification of larval tracks. It is also possible, that in spite of the care taken to maintain the temperature of agar plates within the tolerance range of *S. stercoralis* larvae, some larval death may have occurred during transport. The sensitivity of the direct smear microscopy may have also been high due to large numbers of parasites present and/or an experienced microscopist.

As collection, processing and analysis of fecal samples presents a number of logistical challenges, we undertook three different methods for the diagnosis of *S. stercoralis* that might be more suited to studies in remote field sites. PCR on DNA extractions of ethanol-preserved fecal samples was at least as sensitive as agar plate culture and formalin sedimentation. PCR has the advantage of reducing the handling of fecal specimens in the field, and allowed samples to be batched for testing. This method could be modified to have greater utility in remote field settings. Alternative methods of fecal preservation that allow storage at room temperature [34] could be used, avoiding the need for cold storage. Methods utilizing loop-mediated isothermal amplification (LAMP) require only a single temperature incubation and have the possibility of incorporating visualization of positive results by color or turbidity change [35,36] that could be conducted in a field laboratory. Detection of multiple intestinal parasite species can also be achieved with PCR, using multi-parallel [37] or multiplexed reactions [38,39]. The sensitivity of DNA amplification methods may further be improved by targeting high copy number sequences identified by whole genome sequence analysis [40].

Serology was positive for 16.2% of samples in 2010–2011, higher than for the other diagnostic methods used, and consistent with previous reports [9,41]. In endemic areas, serology has a high positive predictive value due to the high prevalence of infection [8] but may not identify all acute infections, and may be of most use in monitoring the effect of treatment in an individual or population over time [6,19].

## 5. Conclusions

PCR of fecal samples and serological testing of dried blood spots were shown to be at least as sensitive as microscopy and culture for the diagnosis of *S. stercoralis* in this setting. These methods have considerable advantages for use in remote field studies and may be useful for the ongoing assessment of efforts to reduce the prevalence of *S. stercoralis* in remote communities.

Despite a reduction in the percentage of children aged <10 years positive for *S. stercoralis,* hookworm and *T. trichiura* in 2010–2011 compared to 1994–1996 in this remote Aboriginal community in Australia, the rates of *S. stercoralis* and *T. trichiura* remained high at 4.7% and 48.2% respectively. The percentage of children positive for *R. nana* was unchanged. The reduction is consistent with public health measures in the region to reduce intestinal helminths but requires further investigation to assess the impact that these infections are having on child health and development.

## Figures and Tables

**Table 1:** Intestinal parasites identified in fecal samples by direct smear microscopy.

n	1994–1996 84	2010–2011 85	Fisher Exact Probability Test *p* (Two-tail)
Average age (years)	5.6	3.7	
*Strongyloides stercoralis*	11 (13.1%)	4 (4.7%)	0.063
Hookworm	11 (13.1%)	1 (1.2%)	0.002 *
*Rodentolepis nana*	20 (23.8%)	19 (22.4%)	0.857
*Trichuris trichiura*	57 (67.9%)	41 (48.2%)	0.012 *

* *p* < 0.05.

**Table 2:** Comparison of *Strongyloides stercoralis* diagnostic methods in children aged <10 years in 2010–2011.

Method	+ve/n (%)
Direct smear	4/85 (4.7%)
Culture and formalin sedimentation	5/77 (6.5%)
*S. stercoralis* 18S rDNA PCR	6/83 (7.2%)
Serology on dried blood spots	25/154 (16.2%)
